# Changes in adult cannabis use patterns following retail availability in California (2017 to 2024)

**DOI:** 10.1186/s42238-026-00475-z

**Published:** 2026-08-01

**Authors:** Jamie Corroon, Gretchen Bandoli, Igor Grant

**Affiliations:** 1 Department of Psychiatry, Center for Medicinal Cannabis Research, University of California, San Diego, La Jolla, CA USA; 2 Herbert Wertheim School of Public Health and Human Longevity Science, University of California, San Diego, La Jolla, CA USA

**Keywords:** Cannabis, Marijuana, Legislation, Drug, California

## Abstract

**Background:**

California was among the earliest U.S. states to legalize non-medical cannabis use by adults, with retail availability beginning January 1, 2018. This study describes changes in cannabis use prevalence, frequency, and methods among adults in California from 2017 through 2024, spanning the year before and seven years after the onset of retail availability.

**Methods:**

Data were drawn from 178,836 adults aged ≥18 years in the California Health Interview Survey (2017–2024). Survey-weighted logistic regression models with year as a categorical predictor were used to estimate year-specific prevalence, with Tukey adjustment for pairwise comparisons. All analyses accounted for survey weights, strata, and primary sampling units.

**Results:**

Past-year and past-month cannabis use were reported by 24.7% (95% CI: 24.3–25.0) and 16.4% (95% CI: 16.1–16.8) of respondents, respectively. Past-year use was 22.1% (95% CI: 20.9–23.2) in 2017 and 23.5% (95% CI: 22.8–24.2) in 2024 (*p = * 0.24). Past-month use rose from 14.5% (95% CI: 13.5–15.5) in 2017 to 17.7% (95% CI: 16.6–18.8) in 2018 (*p <* 0.01), then fell to 15.4% (95% CI: 14.8–16.0) in 2024, which did not differ significantly from 2017 (*p = * 0.65). Frequency of use was concentrated at the ends of the distribution (1–5 days/month: 40%; 20–30 days/month: 37.3%), with no statistically significant differences across years. Combustible methods (joints, pipes, or bongs) declined from 71.2% (95% CI: 69.2–73.2) to 58.1% (95% CI: 55.8–60.4) (*p <* 0.01), while edible use rose from 40.3% (95% CI: 37.4–43.2) to 53.6% (95% CI: 51.4–55.8) (*p <* 0.01). From 2017 to 2024, past-month use increased among adults aged 40–59 years from 10.7% (95% CI: 9.5–12.1) to 14.5% (95% CI: 13.5–15.6) and among adults ≥75 years from 2.4% (95% CI: 1.5–3.9) to 5.5% (95% CI: 4.6–6.4) (both *p <* 0.01).

**Conclusions:**

Prevalence of past-year and past-month cannabis use among adults in California remained largely stable across the eight-year study period spanning the onset of retail availability of non-medical cannabis products. Frequency of past-month use also remained stable. Methods of use shifted notably, however, with declines in combustible methods and corresponding increases in edible consumption. Past-month use increased among middle-aged and older adults, particularly those aged ≥75 years, while remaining unchanged among young adults (18–25 years).

**Supplementary Information:**

The online version contains supplementary material available at https://doi.org/10.1186/s42238-026-00475-z.

## Introduction

Over the past decade, cannabis policies in the United States have undergone rapid and significant changes. As of 2024, most states permitted medical cannabis use, an increasing number had legalized non-medical use by adults aged 21 years and older (Hasin and Walsh 2021; Cerdá et al. 2008). In fact, more than half of the U.S. population lived in states where non-medical use by adults was legal (National Conference of State Legislatures 2026), despite non-medical use remaining prohibited under U.S. federal law (Drug Scheduling 2026). Additionally, the 2018 Farm Bill further complicated the regulatory landscape by increased availability of intoxicating hemp-derived products outside state-licensed cannabis dispensaries (Governor Newsom 2025; Babalonis et al. 2021). These shifts raise important public health questions about how cannabis use patterns have evolved alongside expanding legalization and retail availability.

California, the most populous state and among the earliest adopters of cannabis policy reforms, offers a unique opportunity to study these changing patterns. California first legalized cannabis for medical use in 1996 with the Compassionate Use Act (Proposition 215), allowing qualified patients to possess and cultivate cannabis for personal use (California Code 2026). Subsequently, California legalized non-medical cannabis for adults in November 2016 under the Adult Use of Marijuana Act (Proposition 64), with commercial sales beginning January 1, 2018 (California Office of the Secretary of State 2016).

Existing evidence on patterns of cannabis use following legalization and retail availability on non-medical use is mixed and varies by geography, age, and other demographic factors (Pacula and Smart 2017). When compared to states without it, U.S. states with legalized non-medical cannabis generally exhibit higher overall cannabis use prevalence than those without legalization, although such differences may have preceded legalization in some states (Hasin et al. 1991). At the state level, early adopters such as Colorado, Washington, Alaska, and Oregon have reported increased use among adults, but stable or even decreased use among adolescents (Oregon Health Authority 2019; SEOW 2023; MPP 2025). Recent data from the U.S. National Survey on Drug Use and Health (NSDUH) suggest national increases in self-reported past-year cannabis use and days of use per capita between 2008 and 2022 (Caulkins 2024). A separate NSDUH analysis of recreational marijuana legalization (2008 to 2016) reported that frequent use increased among adults aged 26 years or older in the overall population but remained unchanged among past-month users in the same age group, suggesting the increase reflected a pattern of more adults using cannabis rather than heavier use among existing users, (Cerdá et al. 2008) an interpretation supported by another NSDUH analysis (2008–2019) that found increased cannabis-use initiation among adults older than 21 years following legalized retail sales (Montgomery et al. 2022). In California, legalization of medical cannabis in 1996 was associated with increased use by adults (Lloyd and Striley 2018). Few population-level studies have evaluated changes in cannabis use patterns in California following legalization and subsequent onset of retail availability however. Prior California Health Interview Survey (CHIS)-based analyses have characterized cannabis use patterns (ever, current, past-month) among young adults in 2018 (Meng and Ponce 2020), among Baby Boomers, from 2017 to 2018 (Carlson et al. 2022), and across racial/ethnic groups from 2018 to 2023 (Caetano et al. 2026). However, these studies have been limited by either a short post-retail observation window or a focus on selected age or racial/ethnic subgroups.

The present study extends this prior work by providing a comprehensive descriptive account of adult use in California from 2017 through 2024, spanning the year before and seven years after the onset of retail availability. Using CHIS, the largest state-level health survey in the United States, (UCLA Center for Health Policy Research 2024) and beginning with the earliest year for which cannabis-use measures were available, we characterize prevalence, frequency, methods of use, and sources of procurement, both overall and across age, sex, and racial/ethnic subgroups. This descriptive analysis describes how patterns of use have evolved in the nation's most populous state during a period of significant cannabis policy reform.

## Methods

### Study population

The CHIS is the largest state-level health survey in the United States, conducted by the University of California, Los Angeles (UCLA) Center for Health Policy Research (CHPR). (UCLA Center for Health Policy Research 2024) CHIS is a continuous, population-based survey that used computer-assisted telephone interviews (CATI) with a dual-frame random-digit-dialing (RDD) design prior to 2019, and since 2019 has employed an address-based sampling (ABS) design with mixed web and telephone administration to achieve representation from all 58 counties in California. Within sampled households, one adult respondent is randomly selected. CHIS adult unconditional response rates ranged from 3.4% in 2017–18 to 7.4% in 2023–24 (CHIS 2019, 2025) Population weights are incorporated based on State of California Department of Finance estimates. These weights address unequal sampling probabilities and non-response biases inherent in complex survey designs. Although item missing rates during data collection ranged from 0.5% to 5.6%, variables do not contain missing values, as CHIS imputes values when respondents do not provide a valid response. The analysis of de-identified, publicly available data was determined to be exempt from IRB review.

### Independent variables

#### Cannabis use

Cannabis use was evaluated using CHIS data collected from 2017 to 2024. Ever use (2017–2024) was assessed by responses to the question, “Have you ever tried marijuana or hashish?”, categorized as yes/no. Past-month use (2017–2024) was determined by the question, “How long has it been since you last used marijuana or hashish?”; respondents whose last use was within the past month were classified as past-month users. Frequency of use (2018–2024) was evaluated among past-month users, starting in 2018 for respondents aged 18–25 years and expanding to all respondents from 2019 onward. Respondents were asked, “During the past 30 days, on how many days did you use marijuana, hashish, or THC products?”, with initial responses categorized as 0, 1–2, 3–5, 6–9, 10–19, 20–29, or 30 or more days, then further collapsed into categories: 1–5 days, 6–9 days, 10–19 days, and 20–30 or more days. Methods of use (2018–2024) were assessed among past-month users, initially among respondents aged 18–25 years in 2018 and among all respondents from 2019 onward. Methods included smoking (joint, bong, pipe, blunt), vaporizing, dabbing, consuming edibles, or drinking cannabis-infused beverages, each categorized as yes/no. Sources of procurement (2021–2024) were assessed among past-month users and included dispensary, smoke shops, other shop, delivery, web, pop-up, family/friends, other person, homegrown, and other sources, each categorized as yes/no (Supplementary Materials).

#### Sociodemographic and other characteristics

Self-reported age was reported as a categorical variable for all survey years and analyzed as five age group categories (18–25, 26–39, 40–59, 60–74, and 75+ years) to facilitate interpretation and ensure adequate sample sizes within strata.

Race and ethnicity were defined using the unabridged classification developed by UCLA CHPR. This variable assigns respondents to mutually exclusive racial/ethnic groups based on self-identification. The original categories included Latino, Other Single/Multiple Race, American Indian/Alaska Native, Asian, African American, and White. For the purposes of this analysis, these categories were collapsed into five groups: Latino; Other/Multiple Races (including American Indian/Alaska Native); Asian; Black/African American; and White.

Education level was categorized into three groups: high school or less, some college (attending some college or vocational school, or attaining a two-year associate degree), and college degree or more. Federal poverty level (FPL) was grouped into three categories: <200%, 200–299%, and ≥300%, representing household income as a percentage of the FPL.

Alcohol use was assessed using two CHIS variables available from 2021 to 2024: 1) Ever had an alcoholic beverage and 2) Number of days drank at least one alcoholic beverage in the past 30 days. Respondents who reported never having consumed an alcoholic beverage were classified as Never/No Past-Month Use. Among those who reported ever drinking, responses to the past-month frequency item were categorized as follows: occasional use (1–4 days), regular use (5–14 days), and frequent use (≥15 days).

Health status was assessed by the question, “Would you say that in general your health is…?” and categorized into poor/fair, good, and very good/excellent.

Cigarette smoking status was determined from self-reported responses and categorized as never, former, or current smoker. Illicit substance use was assessed using four binary (yes/no) items available in CHIS from 2019 to 2024: past-year use of methamphetamines, prescription stimulants not prescribed to the respondent, heroin, and prescription painkillers not prescribed to the respondent. Respondents who responded yes to any of these items were classified as reporting illicit substance use in the past year; all others were classified as non-users.

Nervousness was measured by the question, “During the past 30 days, about how often did you feel nervous?”, originally with responses ranging from “Not at all” to “All of the time”. Depression was similarly measured by the question, “During the past 30 days, about how often did you feel depressed?”.

Psychological distress was measured using the Kessler-6 (K6) scale, a widely used epidemiological tool designed to identify persons with high likelihood of serious mental illness from survey data by measuring the severity of psychological distress. K6 scores range from 0 to 24 and were dichotomized at the established cut point of 13 or higher (See Supplemental Materials) (Carlson et al. 2022).

### Statistical methods

The analytic sample (n=178,836) included respondents who completed cannabis-related questions, drawing from combined annual data files spanning eight years (2017–2024). Person-level sampling weights, strata, and primary sampling units were applied to all analyses to account for the complex survey design. Descriptive statistics were reported as unweighted counts and weighted percentages. For binary outcomes, survey-weighted logistic regression models were fitted with year included as a categorical predictor, and omnibus Wald tests were used to assess whether prevalence differed significantly across years. For multi-category outcomes (e.g., frequency of past-month use), differences in the distribution of responses across years were first assessed using the Rao–Scott F test. For follow-up comparisons of specific frequency categories, each category was analyzed as a separate binary outcome indicating membership in that category versus all other frequency categories. Survey-weighted quasibinomial regression models with year specified as a categorical predictor were used to compare the predicted prevalence of each category across years. Marginal means and Tukey-adjusted pairwise contrasts between years were estimated from these models. Models used a quasibinomial family to obtain robust variance estimates in the presence of potential overdispersion. All analyses were performed in R (Version 4.5.2).

## Results

### Cannabis use patterns

From 2017 to 2024, an estimated 24.7% (95% CI: 24.3–25.0) of adults reported past-year cannabis use and 16.4% (95% CI: 16.1–16.8) reported past-month use (Table 1). Past-month use was most common among adults aged 18–39 years, males, and respondents identifying as non-Hispanic White or Black/African American. Higher prevalence of past-month use was also observed among individuals with at least some college education, those living at ≥300% of the federal poverty level, and those reporting Very Good/Excellent health. Past-month use was more frequently reported among adults who experienced past-month nervousness, depression (All or Most of the Time for both), or psychological distress, those reporting current cigarette smoking, and regular or frequent alcohol use.

**Table 1:** Characteristics of Adults in California by Cannabis Use Status

**California Health Interview Survey (2017 to 2024)**					
		**Cannabis Use Status**			
	**Overall Sample** **178,836 (100.0)**	**Never Use** **81,988 (50.5)**	**Prior Use, No Past-Month Use** **67,761 (33.1)**	**Past-Month Use** **29,087 (16.4)**	***p***-value
Socio-demographics					
Age, years, n (%)					<0.01
18–25	11,008 (14.5)	5,266 (50.2)	2,926 (25.7)	2,816 (24.1)	
26–39	28,932 (24.6)	11,279 (42.4)	10,921 (35.5)	6,732 (22.2)	
40–59	57,833 (31.9)	26,307 (52.9)	22,173 (33.4)	9,353 (13.7)	
60–74	56,118 (20.0)	22,640 (47.8)	24,847 (39.2)	8,631 (13.1)	
75+	24,945 (8.9)	16,496 (70.5)	6,894 (23.8)	1,555 (5.7)	
Sex, n (%)					<0.01
Female	100,310 (51.1)	48,520 (54.5)	37,258 (31.4)	14,532 (14.1)	
Male	78,526 (48.9)	33,468 (46.3)	30,503 (34.9)	14,555 (18.8)	
Race/Ethnicity, n (%)					<0.01
Latino	24,221 (23.0)	14,052 (60.9)	7,076 (26.5)	3,093 (12.6)	
Other/Multiple Races	23,020 (15.9)	10,880 (51.6)	8,090 (30.9)	4,050 (17.5)	
Asian	23,060 (14.8)	17,033 (73.5)	4,383 (18.6)	1,644 (7.9)	
Black/African American	8,343 (5.7)	3,702 (44.5)	3,028 (34.3)	1,613 (21.1)	
White	100,192 (40.6)	36,321 (36.5)	45,184 (42.9)	18,687 (20.7)	
Education, n (%)					<0.01
High School or Less	34,743 (37.0)	19,624 (61.1)	9,883 (24.8)	5,236 (14.1)	
Some College	50,068 (21.2)	21,568 (44.7)	19,374 (36.3)	9,126 (19.0)	
College Degree or More	94,025 (41.8)	40,796 (43.9)	38,504 (38.8)	14,725 (17.3)	
Federal Poverty Level, n (%)					<0.01
< 200%	44,584 (30.9)	23,886 (61.4)	13,114 (23.9)	7,584 (14.7)	
200–299%	22,022 (12.9)	10,728 (53.1)	7,686 (30.8)	3,608 (16.1)	
>=300%	112,230 (56.2)	47,374 (43.9)	46,961 (38.7)	17,895 (17.5)	
Health Status, n (%)					
General Health					<0.01
Poor/Fair	29,434 (17.7)	14,508 (55.8)	9,885 (28.2)	5,041 (15.9)	
Good	54,535 (31.2)	25,730 (52.0)	19,822 (32.0)	8,983 (16.0)	
Very Good/Excellent	94,867 (51.1)	41,750 (47.7)	38,054 (35.4)	15,063 (16.9)	
Mental Health (Past-Month), n (%)					
Felt Nervous					<0.01
All or Most of the time	13,213 (9.1)	4,471 (38.1)	5,035 (33.5)	3,707 (28.4)	
Some of the time	39,649 (24.6)	15,935 (45.5)	15,483 (34.1)	8,231 (20.4)	
A little of the time	61,121 (33.4)	26,887 (49.4)	24,656 (35.4)	9,578 (15.3)	
Not at all	64,768 (32.9)	34,637 (58.7)	22,564 (29.9)	7,567 (11.4)	
Felt Depressed					<0.01
All or Most of the time	4,884 (3.2)	1,760 (39.4)	1,763 (32.9)	1,361 (27.7)	
Some of the time	13,284 (8.8)	5,306 (45.0)	4,784 (31.5)	3,194 (23.5)	
A little of the time	26,114 (16.3)	11,162 (48.2)	9,444 (31.0)	5,508 (20.8)	
Not at all	134,469 (71.6)	63,702 (52.1)	51,747 (33.8)	19,020 (14.1)	
Psychological Distress					<0.01
Yes	9,612 (6.9)	3,151 (37.5)	3,494 (32.4)	2,967 (30.1)	
No	169,139 (93.1)	78,779 (51.4)	64,244 (33.2)	26,116 (15.5)	
Lifestyle & Behavioral Factors					
Cigarette Smoking Status					<0.01
Current	12,475 (7.2)	2,972 (26.9)	5,154 (36.6)	4,349 (36.5)	
Former	44,877 (19.9)	12,439 (30.1)	22,958 (49.0)	9,480 (21.0)	
Never	121,484 (73.0)	66,577 (58.3)	39,649 (28.4)	15,258 (13.2)	
Alcohol use (Past-Month)					<0.01
Never/No Past-Month Use	39,679 (46.6)	24,790 (68.7)	11,020 (22.3)	3,869 (9.1)	
Occasional Use (1–4 days)	22,325 (24.5)	9,524 (44.4)	8,916 (36.9)	3,885 (18.7)	
Regular Use (5–14 days)	15,545 (16.2)	4,789 (31.9)	7,054 (41.6)	3,702 (26.5)	
Frequent Use (>=15 days)	14,848 (12.8)	3,630 (25.8)	7,345 (46.6)	3,873 (27.6)	
Illicit Substance Use (Past-Year)					<0.01
Yes	3,722 (3.0)	867 (26.3)	1,446 (35.3)	1,409 (38.4)	
No	132,784 (97.0)	61,826 (52.1)	49,963 (32.0)	20,995 (15.9)	

Unweighted counts and weighted percentages (row percentages).

Prior Use, No Past-Month Use denotes respondents who reported ever using cannabis but not within the past month.

*p*-values calculated via Rao-Scott Chi-Square test.

Alcohol use data only available for 2021-2023.

Illicit Substance Use data only available for 2019-2023; queried substances were past-year use of methamphetamines, prescription stimulants not prescribed to the respondent, heroin, and prescription painkillers not prescribed to the respondent.

FPL - income as a percentage of the federal poverty level (FPL).

### Cannabis use characteristics of past-month users

Frequency of use displayed a bimodal distribution, with use concentrated at both the lower (1–5 days) and upper (≥20 days) ends of the distribution (~40% each) (Table 2). An estimated 63% reported joint, pipe, or bong as the most frequent method of use, and a dispensary as the most frequent source of procurement (~77%)

**Table 2:** Cannabis Use Characteristics of Past-Month Users (Ages 18+)

*California Health Interview Survey (2018 to 2024)*	
**Characteristic**	**n (%)**
**Frequency**	
1–5 days	7,722 (40.0%)
6–9 days	1,746 (9.0%)
10–19 days	2,687 (13.7%)
20–30 days or more	7,357 (37.3%)
**Methods**	
Joint, Pipe, or Bong	11,764 (62.9%)
Edible	10,004 (51.1%)
Vape	6,094 (37.8%)
Blunt	2,136 (17.2%)
Dab	1,657 (12.6%)
Drink	1,407 (8.0%)
**Sources**	
Dispensary	10,201 (77.4%)
Family/Friend	3,165 (23.4%)
Delivery	1,373 (12.9%)
Homegrown	1,134 (5.5%)
Other Person	596 (4.4%)
Smoke Shop	237 (2.6%)
Web	255 (2.3%)
Pop-Up	108 (1.0%)
Other Shop	97 (0.9%)
Other	32 (0.3%)
	7,722 (40.0%)

Unweighted counts and survey-weighted percentages among past-month cannabis users.

Frequency and methods-of-use items were available for respondents aged 18–25 years in 2018 and for all adult respondents from 2019 onward; all-adult frequency summaries exclude 2018.

Sources of procurement were available from 2021 onward.

### Changes in cannabis use over time

**Fig. 1:**
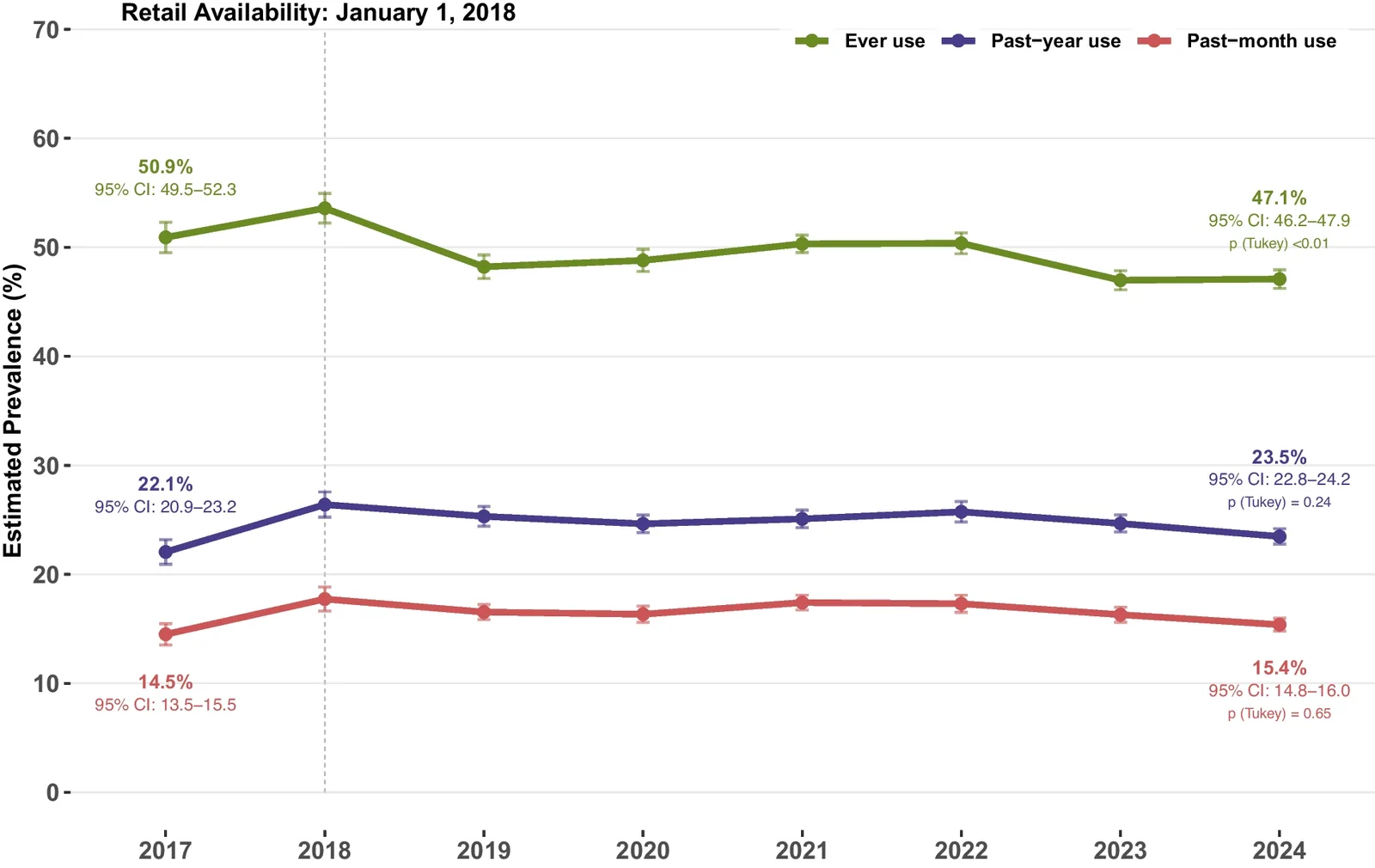
Cannabis Use Patterns. California Health Interview Survey (2017 to 2024). Note: Survey−weighted prevalence estimates with 95% confidence intervals. Tukey−adjusted pairwise comparisons: 2024 v. 2017

Prevalence of ever, past-year, and past-month cannabis use differed significantly across years (omnibus Wald tests, all *p <* 0.01). Ever-use prevalence decreased from 50.9% (95% CI: 49.5–52.3) in 2017 to 47.1% (95% CI: 46.2–47.9) in 2024 (*p <* 0.001; Fig. 1). Past-year use fluctuated over the study period but ultimately did not differ significantly between 2017 (22.1%; 95% CI: 20.9–23.2) and 2024 (23.5%; 95% CI: 22.8–24.2) (*p = * 0.24). Past-month use initially rose from 14.5% (95% CI: 13.6–15.5) in 2017 to 17.7% (95% CI: 16.7–18.9) in 2018 (*p <* 0.01), then remained relatively stable through 2023 before dropping to 15.4% (95% CI: 14.8–16.0) in 2024, which did not differ significantly from 2017 (*p = * 0.65).

### Frequency of Use

**Fig. 2:**
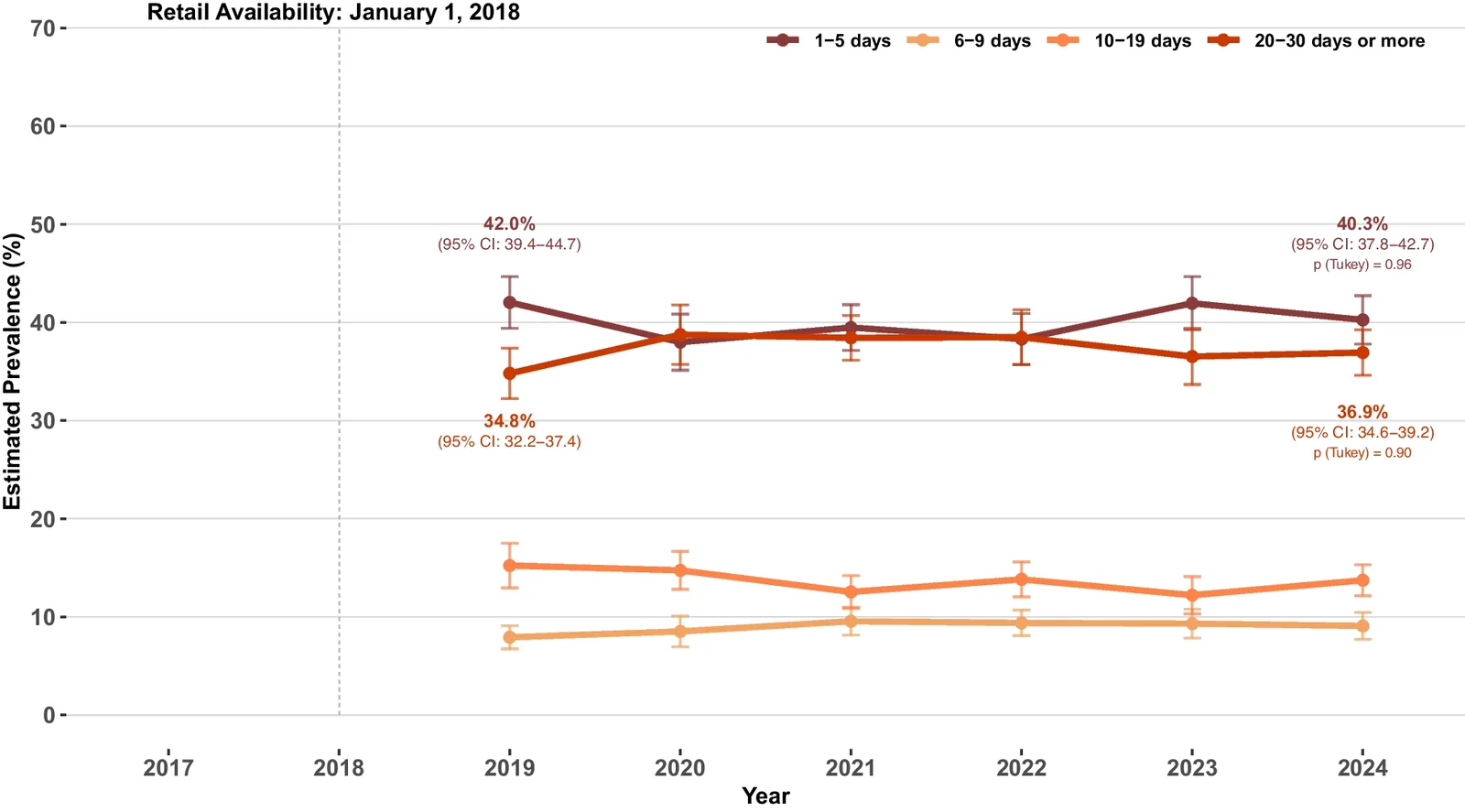
Frequency of Past-Month Cannabis Use Among Past-Month Users. California Health Interview Survey (2019−2024). Note: Survey−weighted prevalence estimates with 95% confidence intervals. 2018 excluded (age−restricted sample).Tukey−adjusted pairwise comparisons: 2024 to 2019.To improve readability, prevalence, 95% confidence intervals, and Tukey−adjusted p−values are annotated for selected frequencies only

Among those reporting past-month use, the distribution of use-frequency was stable across years (2019–2024) (Rao–Scott F test, *p = * 0.44) (Fig. 2). Occasional use (1–5 days) was reported by 42.0% (95% CI: 39.4–44.7) of respondents in 2019 versus 40.3% (95% CI: 37.8–42.7) in 2024 (*p = * 0.96). Similarly, frequent use (20–30 days or more) was 34.8% (95% CI: 32.3–37.4) in 2019 and 36.9% (95% CI: 34.6–39.2) in 2024 (*p = * 0.90). No statistically significant differences across years were observed for moderate-frequency use (6–9 or 10–19 days per month).

### Methods of Use

**Fig. 3:**
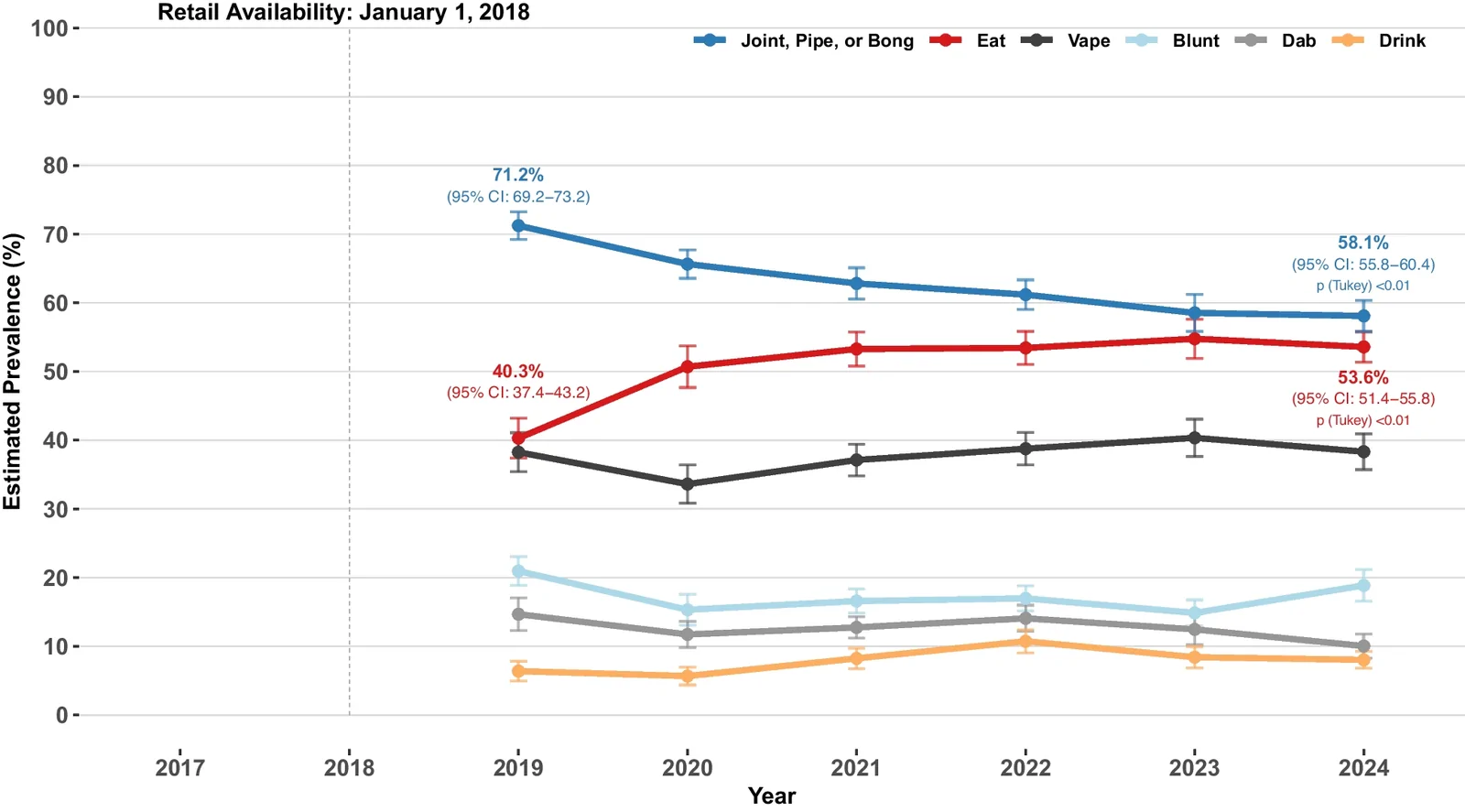
Methods of Cannabis Use by Year Among Past−Month Users. California Health Interview Survey (2019−2024). Note: Survey−weighted prevalence estimates with 95% confidence intervals. Items are not mutually exclusive. 2018 excluded (age−restricted sample).Tukey−adjusted pairwise comparisons: 2024 to 2019.To improve readability, prevalence, 95% confidence intervals, and Tukey−adjusted p−values are annotated for selected methods only

Methods of cannabis use among past-month users were compared from 2019 through 2024, reflecting the period during which methods of use data were available for all adults. The use of joints, pipes, or bongs declined from 71.2% (95% CI: 69.2–73.2) to 58.1% (95% CI: 55.8–60.4) in 2024 (*p*<0.01), while edible use increased from 40.3% (95% CI: 37.4–43.2) to 53.6% (95% CI: 51.4–55.8) (*p*<0.01) (Fig. 3). In contrast, vaping did not differ between 2019 and 2024 (38.2% vs. 38.3%; *p > * 0.99), nor did blunt use (21.0% vs. 18.9%; *p = * 0.82). Dabbing showed a marginal decrease from 14.7% to 10.0% (*p = * 0.06).

### Stratified estimates over time

Patterns of past-month cannabis use differed significantly by age group (p for interaction < 0.001). Prevalence increased among adults aged 40–59 years from 10.7% (95% CI: 9.5–12.1) in 2017 to 14.5% (95% CI: 13.5–15.6) in 2024 (*p <* 0.001), and among adults aged ≥75 years from 2.4% (95% CI: 1.5–3.9) in 2017 to 5.5% (95% CI: 4.6–6.4) in 2024 (*p = * 0.005). No statistically significant changes from 2017 to 2024 were observed among adults aged 18–25 years (*p = * 0.453), 26–39 years (*p = * 0.979), or 60–74 years (*p = * 0.289).

Patterns of past-month use did not differ significantly by sex (p for interaction = 0.26). Among males, prevalence rose from 17.1% (95% CI: 15.9–18.3) in 2017 to 21.0% (95% CI: 19.4–22.6) in 2018 (*p <* 0.01), before returning to 17.5% (95% CI: 16.6–18.4) in 2024, which did not differ significantly from 2017 (*p > * 0.99). Among females, prevalence was 12.1% (95% CI: 10.5–13.8) in 2017 and 13.4% (95% CI: 12.6–14.2) in 2024; the two estimates did not differ significantly (*p = * 0.77).

Patterns of past-month use differed significantly by race/ethnicity (p for interaction < 0.001). Among White adults, prevalence rose from 17.8% (95% CI: 16.4–19.4) in 2017 to 20.5% (95% CI: 19.5–21.5) in 2024 (*p = * 0.05). In contrast, no statistically significant changes in past-month use between 2017 and 2024 were observed among Latino adults (2017: 10.3%; 95% CI: 8.7–12.2; 2024: 10.7%; 95% CI: 9.5–12.1; *p > * 0.99), Asian adults (2017: 7.6%; 95% CI: 5.0–11.3; 2024: 6.6%; 95% CI: 5.7–7.6; *p > * 0.99), African American adults (2017: 16.5%; 95% CI: 12.0–22.1; 2024: 22.3%; 95% CI: 19.0–26.0; *p = * 0.61), or adults reporting Other/Multiple Races (2017: 17.3%; 95% CI: 14.6–20.5; 2024: 17.1%; 95% CI: 15.3–18.9; *p > * 0.99).

Taken together, increases in past-month cannabis use over time were concentrated among middle-aged and older adults and among White adults, while prevalence remained largely stable across sex and other racial/ethnic groups.

## Discussion

Using serial cross-sectional data from CHIS, the largest state-level health survey in the U.S., we analyzed cannabis use prevalence and temporal patterns in all 58 counties of California from 2017, one year prior to the onset of retail availability, through 2024, seven years after. Past-year and past-month use were reported by roughly 1 in every 4 (24.7%) and 1 in every 6 (16.4%) adults aged 18 years and older, respectively. Overall, the prevalence of ever-use declined, past-year use did not change, and past-month use rose initially before returning in 2024 to a level that was not different from 2017. These patterns are consistent with an initial increase in past-month use that was not sustained through 2024. Among past-month users, the distribution of use frequency was stable, indicating no increase in light, moderate or heavy use.

The absence of an overall increase in past-month cannabis use may seem counterintuitive in the context of greater retail availability, especially given that perceptions of health benefits of cannabis have increased (Hall and Lynskey 2016) while perceptions of risk have decreased (Hasin and Walsh 2021). The most parsimonious explanation is that cannabis was already widely accessible in California through long-established medical and illicit markets, so past-month use among adults was already common (Report on the Condition and Health of the Cannabis Industry - Department of Cannabis Control 2026, Substance Abuse and Mental Health Services Administration 2018), leaving comparatively little room for further increases as retail access broadened. Additionally some use may have shifted toward intoxicating hemp-derived products (Whitehill et al. 2024), which may have affected use patterns in ways not captured by CHIS questionnaires.

Temporal changes in past-month use varied across demographic groups. A significant increase was observed among adults aged 40–59 years and those aged 75 or older. The increase among older adults is consistent with other population-level studies and might suggest growing interest in exploring cannabis for health-related purposes (Han et al. 2025; Thayer et al. 2026). At the other end of the age spectrum, past-month use among young adults (18–25 years) did not change, which may offer some reassurance that legalization and retail availability have not been accompanied by increased use in this vulnerable subgroup. Although it should be noted that the 18–25-year age category combines adults who could legally purchase cannabis in California with those aged 18–20 years who could not, limiting our ability to assess differences between these groups. Future research should build on existing studies to determine whether this finding also holds for younger adult and adolescent use. The decline in ever-use may reflect demographic replacement, as cohorts with different lifetime cannabis exposure histories enter and leave the adult survey population.

Our finding of no change in frequency of use is consistent with evidence suggesting that population-level increases in cannabis use following legalization may reflect more adults using cannabis rather than more frequent use by existing users (Cerdá et al. 2008; Montgomery et al. 2022). National data indicate increases in days of cannabis use per capita over time (2008–2022), while a separate legalization-focused NSDUH analysis found that frequent use increased at the population level among adults aged 26 years or older, but not among past-month cannabis users (2008–2016) (Cerdá et al. 2008; Caulkins 2024). Similarly, data from the Behavioral Risk Factor Surveillance System (BRFSS; 2016-2023) suggest that recreational cannabis legalization was associated with increased use among groups with historically lower use, rather than increasing frequency among those who already use cannabis (2016–2023) (Hawkins et al. 2026). This distinction is important because population-level increases in frequent use measures may occur even when the distribution of frequency among users remains stable.

While joint, pipe, or bong was the most frequently reported method of use (~63%), overall use of inhalation methods declined, except for vaping, while edible use increased. The shift away from inhaled methods to edible methods may reflect perceptions of both the detrimental effects of smoking and the increasing availability of edible products in the legal market. It could be argued that a shift away from smoking cannabis, or inhalation in general, could mitigate certain health risks linked to the byproducts of combustion and other particulate matter (Gieringer 2001). Dispensaries were the most frequently reported source of procurement (~77%) and remained unchanged.

These findings are consistent with broader evidence within the U.S. that cannabis product diversification and retail availability have been accompanied by greater use of non-combustible methods of use, particularly edible or drinkable products. In a 2017–2023 BRFSS analysis, Hawkins et al. reported that eating/drinking cannabis increased after recreational legalization and retail sales, whereas smoking declined yet remained the most prevalent method (Hawkins et al. 2026). Earlier BRFSS data similarly showed that multimodal use was common and that edibles, vaping, and dabbing frequently occurred alongside smoking among past-month users (Schauer et al. 2020; Zerleen et al. 2022).

Three prior studies using CHIS data investigated cannabis use patterns before and after the onset of retail availability. A previous study reported an increase in cannabis use among young adults (ages 18–25) from 23.9% in 2017 to 28.5% in 2018 (Meng and Ponce 2020), aligning with our results for the same timeframe. Our analysis showed a slight, but significant decrease in this age group from 2018 through 2024, however, such that prevalence in 2024 did not differ significantly from 2017. Another study of Baby Boomers (born 1946–1964 and 59 to 77 years old in 2024) reported that cannabis use did not change among this generation from 2017 to 2018, but did increase among non-Baby Boomers (Carlson et al. 2022). These findings are in contrast with our results, which indicated increases among adults aged 40–59 years and aged ≥75 years. Importantly, these studies utilized a short post-legalization or retail availability timeframe (through 2018), when cannabis retail infrastructure was still developing. State-level data from BRFSS for a comparable period indicate increased prevalence of past-month use in California in the years after legalization and leading up to retail availability (2016: 11.0% [95% CI: 10.0–12.1]; 2018: 14.9% [95% CI: 14.0–15.9]) (Uddin et al. 2016). A third study using data from the CHIS also reported no change in past-month use (between 2018 and 2023) and similarly reported increased prevalence among White adults and decreased prevalence among Latino adults (Caetano et al. 2026).

Not all studies agree, however. Studies of the first four states to legalize cannabis for non-medical purposes (Colorado, Washington, Alaska, and Oregon), have reported slight increases in past-month use and frequent past-month use among adults 26 years or older, but no increases among those 18 to 25 years (Hasin and Walsh 2021). Prevalence of cannabis use among young adults (18 to 25 years) in Washington reportedly increased since legalization (2014 to 2019) with the change being mostly driven by those aged 21 to 25 years (Kilmer et al. 2022; Substance Abuse and Mental Health Services Administration 2019). Other data from Colorado and Washington suggest no increases in prevalence before and after legalization, particularly among younger populations (Brooks-Russell et al. 2013; Cerdá et al. 2017; Harpin et al. 2018; Jones et al. 2018; Mason et al. 2016). Our findings in California are consistent with reports from Colorado, Oregon, and Alaska, but contrast with Washington, where increases among young adults, particularly those aged 21–25, have been reported. Recent data from the U.S. NSDUH suggest national increases in use, which coincide with state-level legalization efforts (Caulkins 2024).

Several limitations warrant consideration. The serial cross-sectional design limits causal inference; any observed changes cannot be attributed to any specific policy mechanism. The study period also includes two important period-specific factors that may affect interpretation of temporal changes. First, the 2019 CHIS sampling and mode transition may affect comparability of year-specific estimates around that transition. Because the initial increase in past-month use occurred before the 2019 redesign and the subsequent plateau occurred afterward, some portion of this pattern may reflect survey-mode or sampling-related discontinuity rather than behavioral change alone. Second, the study period includes the COVID-19 pandemic and associated social and economic disruptions, which may have influenced cannabis use patterns independently of the regulatory environment. Furthermore, statewide estimates obscure substantial heterogeneity in local policy environments: a recent report from the California Department of Cannabis Control reported that 54% of California jurisdictions prohibit commercial cannabis activity and 57% prohibit any retail businesses (Report on the Condition and Health of the Cannabis Industry 2026). Consequently, statewide changes reflect a mix of exposure to, and absence of, legal retail access. Cannabis use was self-reported and may be subject to social desirability and recall biases.CHIS adult response rates were also low, raising the possibility of nonresponse bias; while CHIS applies population weights to address unequal sampling probabilities and nonresponse, residual bias in prevalence and changes over time cannot be ruled out. A key strength is the use of CHIS, the largest state-level health survey in the United States, with a representative sample drawn from all 58 California counties across an eight-year window spanning the onset of retail availability. Additional strengths include the availability of data on frequency and methods of use, as well as sources of procurement.

## Conclusion

During the eight-year study period, spanning one year before through seven years after the onset of retail availability of non-medical cannabis products, overall statewide prevalence and frequency of past-month cannabis use among adults in California remained largely stable. Important shifts have occurred within specific demographic subgroups, however, with increased use among those aged 75 and older. While frequency of past-month use remained concentrated at the extremes of occasional (1–5 days) and frequent (≥20 days) use, methods of use shifted, with significant declines in traditional combustible methods (joints, pipes, or bongs) and a corresponding increase in edible use, paralleling the diversification of product offerings in the legal market. Among young adults, cannabis use patterns and frequency have remained stable.

## Supplementary Information

Supplementary Material 1.

## Data Availability

The datasets analyzed are publicly available in the California Health Interview Survey (CHIS) repository, https://healthpolicy.ucla.edu/chis.

## Abbreviations

ABS: Address-based sampling
BRFSS: Behavioral Risk Factor Surveillance System
CATI: Computer-assisted telephone interviews
CBD: Cannabidiol
CHIS: California Health Interview Survey
CHPR: Center for Health Policy Research
CI: Confidence interval
CUD: Cannabis use disorder
IRB: Institutional Review Board
K6: Kessler-6 (psychological distress scale)
RDD: Random-digit-dialing
SUD: Substance use disorder
THC: Tetrahydrocannabinol
UCLA: University of California, Los Angeles
YOA: Years of age
