# Supplementary material for: Changes in adult cannabis use patterns following retail availability in California (2017 to 2024)
Source: J Cannabis Res. 2026 Aug 1;8:115. doi: 10.1186/s42238-026-00475-z (PMC13721487; doi:10.1186/s42238-026-00475-z)
Supplement: Supplementary file 1 [file 42238_2026_475_MOESM1_ESM.docx]

Changes in Adult Cannabis Use Patterns Following Retail Availability in California (2017 to 2024)

Jamie Corroon, ND, MPH^1^

Gretchen Bandoli, PhD^2^

Igor Grant MD, FRCP(C)^1^

^1^ Center for Medicinal Cannabis Research, Department of Psychiatry, University of California, San Diego, La Jolla, CA, USA

^2^ Herbert Wertheim School of Public Health and Human Longevity Science, University of California, San Diego, La Jolla, CA

**Corresponding author**: Jamie Corroon, 310-874-9128, jcorroon@health.ucsd.edu

Table of Contents

[SUPPLEMENTARY METHODS	2](#_Toc219453861)

[Independent Variables	2](#_Toc219453862)

[Methods of Use and Sources of Procurement	2](#_Toc219453863)

[Age	2](#_Toc219453864)

[Mental Health Status	2](#_Toc219453865)

[SUPPLEMENTARY RESULTS	3](#_Toc219453866)

[S. Table 1 - Ever Cannabis Use by Year (2017-2024)	3](#_Toc219453867)

[S. Table 2 - Ever Cannabis Use: Pairwise Differences by Year (2017-2024)	4](#_Toc219453868)

[S. Table 3 – Past-Year Cannabis Use by Year (2017-2024)	5](#_Toc219453869)

[S. Table 4 – Past-Year Cannabis Use: Pairwise Differences by Year (2017-2024)	6](#_Toc219453870)

[S. Table 5 – Past-Month Cannabis Use by Year (2017-2024)	7](#_Toc219453871)

[S. Table 6 – Past-Month Cannabis Use: Pairwise Differences by Year (2017-2024)	8](#_Toc219453872)

[S. Table 7 – Frequency of Past-Month Cannabis Use by Year (2019-2024)	9](#_Toc219453873)

[S. Table 8 – Frequency of Past-Month Cannabis Use: Pairwise Differences by Year (2019-2024)	10](#_Toc219453874)

[S. Table 9 – Methods of Past-Month Cannabis Use by Year (2018-2024)	11](#_Toc219453875)

[S. Table 10 – Methods of Past-Month Cannabis Use: Pairwise Differences by Year (2019-2024)	12](#_Toc219453876)

[S. Table 11 – Sources of Cannabis Procurement Among Past-Month Users (2021-2024)	14](#_Toc219453877)

[S. Table 12 – Sources of Cannabis Procurement Among Past-Month Users (2021-2024)	14](#_Toc219453878)

[S. Figure 1 – Sources of Cannabis Procurement Among Past-Month Users (2021-2024)	15](#_Toc219453879)

[S. Table 14 – Sources of Cannabis Procurement Among Past-Month Users by Year (2021-2024)	16](#_Toc219453880)

[S. Table 15 – Past-Month Cannabis Use by Sex and Year (2017-2024)	18](#_Toc219453881)

[S. Table 16 – Change in Past-Month Cannabis Use by Sex and Year (2024 vs. 2017)	18](#_Toc219453882)

[S. Figure 2 – Past-Month Cannabis Use by Sex and Year (2017-2024)	19](#_Toc219453883)

[S. Table 17 – Past-Month Cannabis Use by Age and Year (2017-2024)	20](#_Toc219453884)

[S. Table 18 – Change in Past-Month Cannabis Use by Age and Year (2024 vs 2017)	20](#_Toc219453885)

[S. Figure 3 – Past-Month Cannabis Use by Age and Year (2017-2024)	21](#_Toc219453886)

[S. Table 19 – Past-Month Cannabis Use by Race/Ethnicity and Year (2017-2024)	22](#_Toc219453887)

[S. Table 20 – Change in Past-Month Cannabis Use by Race/Ethnicity (2024 vs 2017)	22](#_Toc219453888)

[S. Figure 4 – Past-Month Cannabis Use by Race/Ethnicity and Year (2017-2024)	23](#_Toc219453889)

# SUPPLEMENTARY METHODS

# Independent Variables

## Methods of Use and Sources of Procurement

Methods of use (2018–2024) were evaluated among past-month users, initially limited to respondents aged 18–25 years in 2018 and expanded to all respondents from 2019 onward, by responses to the question, “During the past 30 days, how did you use marijuana?”. Respondents indicated whether they used cannabis via smoking (joint, bong, pipe, blunt), vaporizing, dabbing, consuming edibles, or drinking cannabis-infused beverages in the past month, each categorized as Yes or No. Sources of procurement (2021–2024) were evaluated among past-month users, starting in 2021, by responses to the question, “Where did you get the marijuana or cannabis you used in the past 30 days?”, with categories including Dispensary, Smoke Shops, Other Shop, Delivery, Web, Pop-Up, Family/Friends, Other Person, Homegrown, and Other sources, each categorized as Yes or No.

## Age

Self-reported age was reported as a categorical variable for all survey years, with the following groups: 18–25, 26–29, 30–34, 35–39, 40–44, 45–49, 50–54, 55–59, 60–64, 65–69, 70–74, 75–79, 80–84, and 85+ years. For analysis, these categories were collapsed into five broader age groups to facilitate interpretation and ensure adequate sample sizes within strata: 18–25, 26–39, 40–59, 60–74, and 75+ years.

## Mental Health Status

Psychological distress was measured using the Kessler-6 (K6) scale, a widely used epidemiological tool designed to identify persons with high likelihood of serious mental illness from survey data by measuring the severity of psychological distress.^1^ The K6 scores are based on responses to six questions about how often (0 = none of the time to 4 = all of the time) participants felt nervous, hopeless, restless, depressed, worthless, or that everything was an effort in the last 30 days which range from 0 to 24. The final score was dichotomized using the established cut point with scores <13 indicating no psychological distress and scores 13 or above indicating psychological distress.^1^

One K6 item was selected as a proxy for depressive symptoms, “About how often during the past 30 days did you feel so depressed that nothing could cheer you up?”, and another was selected as a proxy for anxiety symptoms, “About how often during the past 30 days did you feel nervous?”, following the approach of Gibb et al.^2^ The five possible responses (never, a little of the time, some of the time, most of the time, all of the time) were collapsed and categorized as: never = reference group; some/a little of the time; and most/all of the time.

# SUPPLEMENTARY RESULTS

## S. Table 1 - Ever Cannabis Use by Year (2017-2024)

## S. Table 2 - Ever Cannabis Use: Pairwise Differences by Year (2017-2024)

## S. Table 3 – Past-Year Cannabis Use by Year (2017-2024)

## S. Table 4 – Past-Year Cannabis Use: Pairwise Differences by Year (2017-2024)

## S. Table 5 – Past-Month Cannabis Use by Year (2017-2024)

## S. Table 6 – Past-Month Cannabis Use: Pairwise Differences by Year (2017-2024)

## S. Table 7 – Frequency of Past-Month Cannabis Use by Year (2019-2024)

## S. Table 8 – Frequency of Past-Month Cannabis Use: Pairwise Differences by Year (2019-2024)

## S. Table 9 – Methods of Past-Month Cannabis Use by Year (2018-2024)

## S. Table 10 – Methods of Past-Month Cannabis Use: Pairwise Differences by Year (2019-2024)

## S. Table 11 – Sources of Cannabis Procurement Among Past-Month Users (2021-2024)

## S. Table 12 – Sources of Cannabis Procurement Among Past-Month Users (2021-2024)

## S. Figure 1 – Sources of Cannabis Procurement Among Past-Month Users (2021-2024)

## S. Table 14 – Sources of Cannabis Procurement Among Past-Month Users by Year (2021-2024)

## S. Table 15 – Past-Month Cannabis Use by Sex and Year (2017-2024)

## S. Table 16 – Change in Past-Month Cannabis Use by Sex and Year (2024 vs. 2017)

## S. Figure 2 – Past-Month Cannabis Use by Sex and Year (2017-2024)

## S. Table 17 – Past-Month Cannabis Use by Age and Year (2017-2024)

## S. Table 18 – Change in Past-Month Cannabis Use by Age and Year (2024 vs 2017)

## S. Figure 3 – Past-Month Cannabis Use by Age and Year (2017-2024)

## S. Table 19 – Past-Month Cannabis Use by Race/Ethnicity and Year (2017-2024)

## S. Table 20 – Change in Past-Month Cannabis Use by Race/Ethnicity (2024 vs 2017)

## S. Figure 4 – Past-Month Cannabis Use by Race/Ethnicity and Year (2017-2024)

**References**

1.	Kessler RC, Andrews G, Colpe LJ, et al. Short screening scales to monitor population prevalences and trends in non-specific psychological distress. *Psychol Med*. 2002;32(6):959-976. doi:10.1017/S0033291702006074

2.	Mitchell CM, Beals J. The utility of the Kessler Screening Scale for Psychological Distress (K6) in two American Indian communities. *Psychol Assess*. 2011;23(3):752-761. doi:10.1037/a0023288
